# The Role of Microbiota in Neutrophil Regulation and Adaptation in Newborns

**DOI:** 10.3389/fimmu.2020.568685

**Published:** 2020-09-29

**Authors:** Trim Lajqi, Johannes Pöschl, David Frommhold, Hannes Hudalla

**Affiliations:** ^1^Heidelberg University Children's Hospital, Department of Neonatology, Heidelberg, Germany; ^2^Klinik für Kinderheilkunde und Jugendmedizin, Memmingen, Germany

**Keywords:** microbiota, trained immunity, neutrophil (PMN), innate immunity, immune priming, newborn - immunology

## Abstract

Newborns are highly susceptible to infections and mainly rely on innate immune functions. Reduced reactivity, delayed activation and subsequent failure to resolve inflammation however makes the neonatal immune system a very volatile line of defense. Perinatal microbiota, nutrition and different extra-uterine factors are critical elements that define long-term outcomes and shape the immune system during the neonatal period. Neutrophils are first responders and represent a vital component of the immune system in newborns. They have long been regarded as merely executive immune cells, however this notion is beginning to shift. Neutrophils are shaped by their surrounding and adaptive elements have been described. The role of “innate immune memory” and the main triangle connection microbiome—neutrophil—adaptation will be discussed in this review.

## The Role of Neutrophils and the Microbiome in Newborns

### Neutrophil Function in Newborns

Neutrophils are innate immune cells which primarily act as first responders to invasive infections. They are the largest subgroup of polymorphonuclear cells (PMNs) and the most abundant type of immune cell in the peripheral blood. Typically, neutrophils constitute around 50% of bloodstream leukocytes in humans, however bacterial infections may trigger an increase of up to 80% in order to eliminate the invading pathogen (1). Neutrophils are terminally differentiated cells mainly characterized by a short lifespan (around 48 h after release into the circulation) (2, 3). They are involved in the defense against bacterial, fungal as well viral infections (4, 5). As first line responder cells, neutrophils harbor an armory of antimicrobial agents including hydrolytic enzymes (i.e., defensins), pro-inflammatory mediators and reactive oxygen species (ROS) (3). They possess the unique capacity to extrude a meshwork of chromatin fibers known as neutrophil extracellular traps (NETs) aiming to eliminate invading pathogens (6). Dysregulations of neutrophil recruitment, activation or survival are closely associated with the pathogenesis of various infectious as well inflammatory diseases (5, 7).

The neonatal immune system must continuously mount immune responses against external or internal factors whilst maturing (8). This immaturity partially accounts for the high mortality from opportunistic infections and renders the first year of life the deadliest until the age of 50 (9). Neutrophils as innate immune cells appear at low counts during gestational week (GW) 8 and reach the peak of expansion around birth (8, 10). The most vulnerable population of extremely premature infants are also born with an immature innate immune system which is less functional compared to term born neonates. Leukocyte recruitment is ontogenetically regulated, whereas extremely premature infants show heavily impaired recruitment, which gradually matures up to around 35 weeks of gestation (11–14). In particular, expression of adhesion molecules of both neutrophils and the endothelium are greatly suppressed in preterm infants as reviewed in detail previously (13). Further, preterm neutrophils show a lower phagocytic capacity toward both gram (+) and gram (–) bacteria compared to both term-born infants and adults, which may be due to reduced levels of opsonization factors such as maternal immunoglobulins (15–18). Neonatal neutrophils have long been considered as primitive and “dysfunctional,” however studies indicate that they show immunological plasticity by adapting in response to environmental cues (18). One such early influence for neutrophil adaptation is the rapidly shifting composition of the perinatal microbiota.

### The Microbiota and the Neonatal Immune System

Early-life events such as the mode of delivery, maternal conditions, the use of antibiotics or pre-/probiotics, diet and many others drive the maturation of the immune system, especially of innate immune cells during infancy (19–22). Such perinatal circumstances also imprint themselves on microbiome composition and can be linked to favorable or adverse immunological outcomes (23). Proving causality between perinatal events, microbiome composition and immunity is difficult. Yet, the so-called “primitive” innate immune system of newborns may make disentangling such correlations a little more feasible.

The microbiota consists of many species including bacteria, viruses, fungi and protozoa. The cell number and the size of the genome of the human microbiota greatly exceeds the host (24). This “second genome” has a profound impact on the way we react to pathogens and how the immune system differentiates between friend and foe (25, 26). The perinatal period is marked as the most dramatic shift in both immune function and microbiome composition. The microbiota rapidly colonizes the newborn and represents a first challenge to the evolving immunity (27–29). Prenatally, a low immunological profile with a tolerogenic phenotype represents a protective intrauterine feature to prevent rejection of the semi-allogenic fetus by the maternal immune system (30–32). After birth, the neonatal immune system must undergo a rapid transition from immune evasion to immune defense. The neonatal immune system learns to “tolerate” its microbiota, however abnormal colonization (dysbiosis) may challenge this adaptation of the host. For example, the mode of delivery is known to prime the neonatal immune system through alterations in microbiome composition (33). Further, vaginal birth triggers the release of many stress hormones such as cortisol and catecholamines that have profound effects on the phenotype and function of a variety of innate immune cells like neutrophils, natural killer (NK) cells and monocytes (8, 22). The excessive use of antibiotics in the neonatal period is another example of microbiome-disruption with lasting consequences and has been linked to the development of chronic diseases (34, 35). Specifically, macrolide antibiotics are known to inhibit the proper activation and recruitment of neutrophils (34, 36). These alterations in the interplay of the microbiota and immune development can significantly impact neonatal morbidity and mortality. Gut dysbiosis is associated with the development of necrotizing enterocolitis (NEC), an often-fatal inflammatory disease of the preterm gut (37). Among very low birthweight infants (<1,500 grams), who are evolutionary not built to handle a postnatal microbiome or to deal with a magnitude of pathogens, alterations in microbiome composition precede the onset of NEC (38). Distortion of intestinal microbiome composition is also discussed as a contributing factor to the development of sepsis (39, 40). Outside immune priming, gut microbiome composition has been shown to influence growth in infants and to impact long-term health trajectories as reviewed in detail by Pflughoeft and Versalovic (41, 42).

### The Interplay Between Microbiota and Neutrophils

Neutrophils as an essential part of the innate immune system are affected by different microbial components or metabolites of both resident microbiota and invading pathogens. Early investigations have shown that gut microbiota may alter the production of neutrophils through modulation of myelopoiesis in the bone-marrow (43–45). Later studies revealed that microbiota depletion leads to increased susceptibility to infections in neonates presumably caused by decreased numbers of neutrophils (40, 45, 46). Microbiome-derived mediators such as IL-17, IL-7, IL-6, stem cell factor (SCF) and thrombopoietin (THPO) affect the release of granulocyte colony-stimulating factor (G-CSF) (40, 47). Interestingly, microbiome alterations occurring by a high-fat diet prevail hematopoiesis by affecting the hematopoietic niche (48, 49). Apart from affecting neutrophil production, the microbiota also regulates neutrophil function. Short-chain fatty acids (SCFAs) as metabolites derived from the gut microbiome, suppress the activation and recruitment of neutrophils (50, 51) and promote the resolution of inflammation through induction of apoptosis in neutrophils and its efferocytosis by macrophages (52, 53). Further, the gut microbiota regulates bile acid metabolism, which have been shown to impact immune cells (54). Data from our lab indicates that tauroursodeoxycholate, a bile acid with chaperoning activity, directly suppresses neutrophil activation and recruitment to inflamed tissue (manuscript under submission). Inversely, neutrophils also have the capacity to modulate the microbiota by removing unwanted species (49). Despite such individual pathways or mediators that may facilitate cross talk between the microbiota and neutrophils, no systematic analysis has been performed for newborns or specifically preterm infants. Despite metabolites, another intriguing hypothesis is the crosstalk between the microbiota and immune cells via extracellular vesicles (EVs) or more specifically exosomes (small EVs with a diameter of 30–150 nm) (55). Whilst there is an ongoing debate as to whether EVs are active signaling components or mainly a “junk disposal system,” it is generally accepted that every living cell produces EVs. The cargo of bacterial EVs depends on the cellular component that they originate from: in Gram-negative bacteria they mainly originate from the outer membrane and contain periplasmic components (outer membrane vesicles, OMVs), whereas Gram-positive bacteria produce bacterial membrane vesicles (BMVs) (56). EVs may contain a variety of cargo including proteins, signaling components, receptors, mRNA and many others. Both OMVs and BMVs are used by bacteria for communication, for example for horizontal transfer of genes of resistance to antibiotics. Further, especially phagocytic immune cells like macrophages or neutrophils may incorporate BMVs or OMVs. For example, OMVs containing small RNA from *P. aeruginosa* can reduce inflammation of airway epithelial cells as well as neutrophil activation and infiltration in murine lungs (57). Also, BMVs from *S. aureus* were shown to exert a pro-inflammatory effect on endothelial cells by upregulating the expression of E-selectin, VCAM-1, ICAM-1 and IL-6 which lead to increased recruitment of monocytes (58). A direct communication of the microbiota with neutrophils via EVs would open the door for interventions or immune modulation using EVs from probiotics or commensal bacteria.

Assuming a direct influence of the microbiota on innate immune function and neutrophils, pre- or probiotics may be a feasible way to fine-tune the innate immune response, especially in preterm infants who are at greater risk for gut dysbiosis. The use of pre- and probiotics as perinatal supplements has been discussed as a possible way to improve the composition of microbiota resulting in favorable short and long-term outcomes (59–61). Specifically Lactobacillus and Bifidobacteria are reported to modulate immune responses acting either as immune activators or immune suppressors (62–65). Furthermore, the probiotic strain *Bifidobacterium longum* 5^1A^ has been reported to decline the pro-inflammatory response, as shown by decreased neutrophil recruitment and accumulation thus improving the ability of mice to deal with lung infections induced by *Klebsiella pneumoniae* (66, 67). Probiotics have been shown to improve neutrophil function and cytokine response in patients with alcoholic cirrhosis and *Lactobacillus rhamnosus* inhibits *Staphylococcus aureus* induced NET formation in mice (68, 69). However, specific reports on the effect of probiotics on neutrophils in newborns are missing. Looking at EVs from bacteria, it was shown that OMVs and BMVs derived from probiotics exert anti-inflammatory effects and promote immune tolerance (70). The advantage of EVs over probiotics could be their relative safety (no living bacteria used, especially in preterm infants), standardization and storage. It would also allow for individualization and gestational-age specific treatment. To date however, there have been no clinical trials for the use of bacteria-derived EVs in humans. As it harbors less implications, we are eager to develop this novel field of research.

## Long-term Adaptation of Neutrophils

Despite direct effects of the microbiota on neutrophil function, the very limited lifespan raises the question whether such alterations by the microbiota have lasting effects on neutrophil populations. The notion of neutrophils as mere effector cells has been challenged and a growing body of evidence suggests that innate immunity is shaped by surrounding factors and displays adaptive elements (71). This new field of immunology is defined as “trained immunity” or “innate immune memory.” This section will explore, how the triangle connection microbiome—neutrophil—adaptation may promote plasticity in the neutrophil population.

### Adaptation—A Twist to Innate Immunity

It has been established that innate immune cells continuously exposed to various pathogens are capable to develop long-term adaptive features manifested with an increase or decrease in their responsiveness. This feature of innate immune cells to mount nonspecific responses displaying thus adaptive characteristics has been defined as memory-like response (72–75). The ability to respond with memory-like (adaptive) behaviors resulting in increased, pro-inflammatory responses after re-challenge by conserved molecules known as pathogen-associated molecular patterns (PAMPs) has been termed as *trained immunity* or *innate memory* (74, 76). So, trained immunity has been defined as a nonspecific immunological memory triggered from long-term functional rewiring of the epigenetic program, evoked by different pathogenic insults that results in an altered response, specifically protection against secondary infections (71, 77). Furthermore, a set of host biomolecules known as danger-associated molecular patterns (DAMPs) have been identified to mount memory-like behaviors in macrophages (78). In contrast, exposure of innate immune cells to gram (–) bacterial lipopolysaccharide (LPS) has been reported to induce endotoxin *tolerance* (desensitization) characterized by decreased pro-inflammation and increased anti-inflammatory responses (79–82).

Initial reports disclosed a PAMP-specific development of innate memory, where priming by β-glucan and Bacillus Calmette-Guerin (BCG), after subsequent challenge by LPS, resulted in increased production of pro-inflammatory cytokines (i.e., IL-1β, TNF-α, IL-6) (83–85). Further studies displayed a pathogen dose-dependent induction of either innate memory by priming with low doses, or tolerance by high doses of sequential challenges with LPS, especially in macrophages and microglia (86–88). Moreover, cellular maturation is another important factor, influencing adaptive features through distinct signaling mechanisms (89, 90). Activation of the phosphoinositide 3-kinase (PI3K)/mechanistic target of rapamycin (mTOR) pathway is critical for the induction of trained immunity in macrophages leading to an increased inflammatory response (91). Contrary, suppression of the mTOR pathway drives immune tolerance, characterized by increased anti-inflammatory responses like IL-10 production and suppression of pro-inflammatory mediators (92). Interestingly, a study found that reduced synthesis of LPS-induced TNF-α is associated with increased activity of AMP-activated protein kinase (AMPK) (93). Several studies further highlighted, that both adaptive features are accompanied by epigenetic reprogramming, with resulting distinct changes in metabolism like increased glycolysis, glutaminolysis or accumulation of fumarate during trained immunity or increased itaconate production during tolerance (91, 94–97). A summary of the metabolic and cellular differences between trained immunity and tolerance is summarized in Table 1.

**Table 1:** Characteristic features of trained immunity and tolerance in innate immune cells.

**Trained immunity**	**Tolerance**	**References**
↑ Pro-inflammatory mediators (such as TNF-α, IL-6, IL-1β, IL-12, ROS)	**↓** Pro-inflammatory mediators (such as TNF-α, IL-6, IL-1β, IL-12, ROS) **↑** Anti-inflammatory mediators (such as IL-10, Arg-1)	(71, 83, 88, 92, 98)
↑ IRAK-1 and/or NF-kB-p65 (RELA)	**↓** NF-kB-p65 **↑** NF-kB-RelB	(88, 99, 100)
Promoted by PI3Ks/mTOR	Promoted by AMPK	(91, 101, 102)
↑ Aerobic glycolysis (increased lactate production) ↓ Fatty acid oxidation (β-oxidation)	**↓** Aerobic glycolysis (decreased lactate production) **↑** Fatty acid oxidation	(71, 91, 103, 104)
Glutaminolysis and accumulation of fumarate	**↑** Production of itaconate	(94, 96, 97)
M1-like phenotype	M2-like phenotype	(105, 106)
↑ Deposition of H3K4me3 or H3K27ac	↑ Deposition of H3K9me3 Regulated by histone methyltransferase G9a	(107–110)

**Figure 1:**
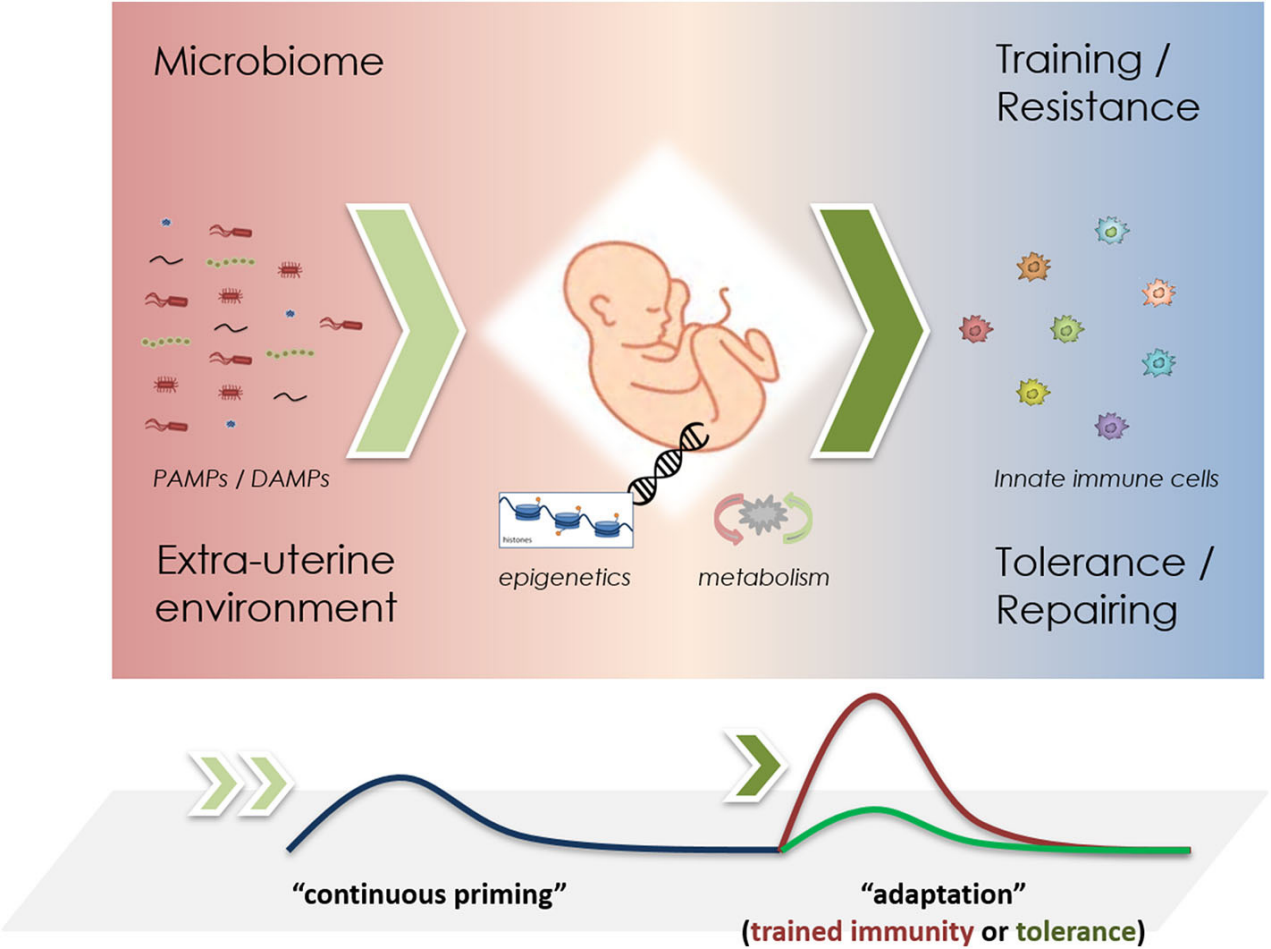
Schematic representation of priming mechanisms in innate immune cells during infancy. The microbiome and the perinatal environment challenge the innate immune system through PAMPs and DAMPs (left), driving epigenetic changes that promote metabolic reprogramming (middle). Upon re-challenge, either trained immunity or tolerance effects may be observed (right). In short-lived neutrophils, it remains unclear, whether “continuous priming” is necessary. If so, a stable microbiome could greatly influence these adaptive responses.

These opposing immuno-inflammatory responses shaped by different external and internal stressors aim at the reduction and elimination of pathogens—trained immunity as resistance mechanism, or are responsible for promoting maintenance and repairing activities in order to facilitate survival—tolerance as a persistence response (101). In newborns, which are largely reliant on their innate immune system, the development of trained immunity may be of crucial importance for the host survival by providing increased protection against pathogens. Figure 1 shows a schematic representation of the development of such adaptive responses in newborns. A recent study expressed the importance of maternal vaccination and the possible training effects of the innate immune system increasing the rate of survival, where children of BCG-vaccinated mothers had for around 35% less hospital admissions for infectious diseases and 41% lower mortality then control groups (111). The crucial role of trained immunity has also been highlighted in physiological processes during pregnancy where NK cells promote the vascular sprouting in the placenta and favor repeated pregnancies (112, 113). Moreover, NK cells have been observed to improve survival rates in BCG vaccinated mice lacking functional B and T cells, stressing the importance of innate memory in NK cells (84). Akin to BCG vaccinations, infants born to mothers with hepatitis B infection are prone to develop trained immunity in neutrophils or other haematopoietic phagocytic cells such as dendritic cells (DCs) (114). Yet, inappropriate induction of these opposing reactions (training vs. tolerance), might provoke maladaptive responses such as hyper-inflammation exhibiting a close relationship to overflowing resistance responses (trained immunity), or increased susceptibility to opportunistic infections (tolerance) (71, 115–117).

### Adaptive Responses in Neutrophils—What Do We Know?

Neutrophils are among the first innate immune cells to enter the site of infection. They are closely shaped by different interactions with the microbiota or the extra-uterine environment (118–121). It was believed that due to their very short live-span neutrophils were unable to participate in enduring memory-like responses (122). However, early investigations showed that neutrophils are prone to be primed by different cytokines, especially IL-8 and TNF-α, improving the recruitment and killing activities of neutrophils in neonates (123, 124). As mentioned above, microbiota-derived mediators may similarly drive adaptive responses (125). For example, microbiome-derived metabolites promote production of antimicrobial peptides such as peptidoglycans that improve the killing capacity of neutrophils (126–128). In line, disruption of the neonatal microbiota by antibiotic exposure is linked to decreased numbers of bone-marrow and peripheral neutrophils, resulting from an impaired granulopoiesis (40).

It is well-known that the pro-inflammatory activity of various innate immune cells increases whilst aging (89, 129, 130). The pro-inflammatory activity of neutrophils characterized by increased tissue infiltration, phagocytosis and NET formation expands with age and is regulated particularly by the TLR4/MyD88 pathway (131). Depletion of the microbiota reduces the number of aged neutrophils and dampens inflammation. The study further confirmed that disruption of the microbiota composition affects the quantity and functional properties of neutrophils. This study outlines the crucial role of microbiota regulating different functional properties of neutrophils, that may drive the inflammatory state toward either resistance mechanisms or a tolerant phenotype. Moreover, microbiota-derived EVs may influence the functional properties of innate immune cells, in particular neutrophils (56, 57, 132, 133). EVs contain a variety of cargo including different proteins, phospholipids, glycolipids, nucleic acids and polysaccharides that are partially able to directly bind to pathogen recognition receptors (PRRs) (132, 134, 135). Furthermore, supplementation by pre- and probiotics may influence the development of adaptive features by neutrophils and other innate immune cells (136–138). Recently, a new concept called “microbiological memory” was introduced aiming to explain the role of microbiome regulating epigenetic rearrangements and their impact on different diseases (139).

A study from Mitroulis et al. showed that trained-immunity-induced effects modulate myeloid progenitors in the bone marrow, especially influencing the recovery of circulating neutrophils thus being of crucial importance for the protection during chemotherapy-induced myelosuppression (140). Furthermore, they revealed that these beneficial effects are closely associated with metabolic changes mainly promoted by altered epigenetic rearrangements. In line with this, another study showed that BCG-induced trained immunity triggers the increase of multipotent and hematopoietic progenitors leading to a sustained myeloid cell expansion (141).

So far it is known that septic reactions in infants are accompanied by a heightened immune activity driven by neutrophils, an energy-demanding process that is mainly promoted by a shift of metabolism toward aerobic glycolysis, a similar process that trained cells undergo (142–144). In line with this, accumulation or activation of several metabolic intermediate products have been shown to either promote the induction of trained immunity (e.g., accumulation of fumarate) or tolerance (i.e., itaconate pathway) thus affecting also main cellular actions of neutrophils that may affect the innate immune response during infancy (94, 97). Priming the niche of neutrophil progenitors may have lasting effects on long-term adaptation. However, the microbiota is a versatile source for innate immune priming, as it may also continuously prime peripheral neutrophils over time (Figure 1).

Taken together, the discovery of the ability of neutrophils to exhibit memory-like responses, open new perspectives for further research in order to understand the impact of trained or tolerized neutrophils in neonatal conditions. A better characterization of the relation duo microbiota—neutrophil could yield potential tailored interventions with probiotics. Here, we are excited to further explore the potential of probiotic-derived EVs as a potentially novel class of safe and standardized biologicals to modulate the immune response. We hope that the PRIMAL Consortium with a clinical trial under way to establish patterns and correlations between gut microbiome and immune profiling may yield answers in the near future (https://primal-studie.de/primal-consortium/).

## Conclusions

Due to its immuno-compromised properties, the neonatal immune system is very susceptible to infectious diseases. Different perinatal factors shape the immunological response of innate immune cells including neutrophils. Through regulation of neutrophil production, function and apoptosis, the microbiota greatly influences the capacity of both initiation and resolution of inflammation. Priming and subsequent induction of trained immunity or tolerance introduces adaptive elements into the innate immune system and allows for immune memory. This concept has been described in detail for many innate immune cells, however only limited insight exists for immune memory in neutrophils and how the microbiota may act as a modulator. Their short lifespan makes neutrophils no easy target to study memory effects and leaves two options by which adaptation may occur: either by continuous priming or by modulating the niche of progenitors in the bone marrow. There is evidence for both, however more studies are needed to shed light on this novel aspect of innate immunity. A better understanding of microbiome—neutrophil interactions (for example through EVs) may support the design of tailored probiotic supplementation in newborns.

## Author Contributions

TL and HH wrote the manuscript. DF and JP revised the manuscript. All authors contributed to the article and approved the submitted version.

## Conflict of Interest

The authors declare that the research was conducted in the absence of any commercial or financial relationships that could be construed as a potential conflict of interest.
